# Short-term health service utilization after a paediatric injury: a population-based study

**DOI:** 10.1186/1824-7288-39-66

**Published:** 2013-10-23

**Authors:** Ileana Baldi, Francesco Avossa, Ugo Fedeli, Francesca Foltran, Dario Gregori

**Affiliations:** 1 Unit of Biostatistics, Epidemiology and Public Health, Department of Cardiac, Thoracic and Vascular Sciences, University of Padova, Via Loredan 18, 35131, Padova, Italy; 2 SER - Epidemiological Department, Veneto Region, Italy, Passaggio Gaudenzio 1, 35131, Padova, Italy

**Keywords:** Injury burden, Childhood, Administrative data

## Abstract

**Background:**

The aim of the study is to identify which types of injuries are responsible for a major component of the health burden in a population-based children cohort in North-Eastern Italy.

**Methods:**

All children (1–13 years) residing in Veneto region, who were hospitalized in 2008 with a International Classification of Diseases, ninth edition, Clinical Modification (ICD-9-CM) code for injury in the first diagnostic field were considered. The outcome was defined as the difference in hospital use in the 12 months following the injury and it was compared to the year preceding the injury occurrence. We computed hospitalization rates by gender, age class and injury type.

**Results:**

Hospitalization rates for injury are highest in males, especially among school-aged children. Rates for intracranial injury exhibit a more pronounced decline with age in females, whereas a more marked rise in upper limb fracture rates among school-aged males is observed. Overall, 3 days of hospital stay per child are attributable to injury. Burns, skull fracture and a high injury severity are associated with a greater number of additional inpatient days.

**Conclusions:**

The impact of specific injury types on health services utilization varies with gender, age and severity. These observed patterns contribute to build a clearer picture of this leading global public health problem and deserve more attention in planning preventive strategies and resource allocation.

## Background

All over the globe, injuries in childhood and adolescence have a major impact on individual and population health causing, even in high-income countries, a high disability and mortality burden. [1] In Europe, each year more than 40,000 children die from injury, and for every child fatality, there are several thousand victims of injury or violence who live with varying degrees of disability or psychological scarring [2,3].

The impact of disproportionately early mortality, the need for critical and costly medical care, and the risk for extended periods of disability, make traumatic injuries a demanding public health and social challenge [4].

While the pattern and aetiology of injuries and their outcomes vary substantially across populations, [5] there is growing awareness of the importance of epidemiological research investigating childhood injury and its determinants, especially those most likely to be amenable to prevention programs.

Data collection across relevant sectors or surveillance systems to quantify the demographic, socioeconomic and epidemiological profile of the burden of child injury is needed to assure that the resources available are commensurate with the extent of the problem.

Burden of disease estimates are increasingly being used to support health-policy decisions relating to clinical, preventive and health services activity [6]. Such estimates differ, though, depending on how information is obtained. On the one hand trauma registries and special surveillance systems contain rich contextual information on particular subsets of injuries, but they cannot be used to estimate the incidence of injury at population level. On the other hand population-based surveys can yield estimates of the total burden of injuries across a broad spectrum of injury severity but they often include insufficient sample sizes for studying small population subgroups and are subject to recall errors [7].

However, thanks to the great improvements in reporting, coding, and classification, administrative health care databases, due to their presumed near complete coverage of injuries requiring medical care and their lack of reliance on self-reports, may be a valuable data source for large population-based studies. Counts and rates of hospitalizations, length of hospital stay (LOS), emergency department visits, admissions to rehabilitation programs and physician services are considered to be valid measures of disease outcomes [8].

The aim of the present study is to identify the injury patterns responsible for a major component of the health burden in a cohort of children in a North-Eastern region of Italy, particularly assessing the difference in hospital use in the 12 months following the injury compared to the year preceding the injury occurrence.

## Methods

### Data sources

In Italy each inpatient case is registered in a national standard hospital discharge form set up by a Ministry of Health decree, summarizing all the clinical information. This form permits the reimbursement of hospital activities on the basis of the Diagnosis Related Groups (DRG) system.

Veneto hospital discharge abstracts cover both hospital stays and day-care of patients hospitalized in Veneto Region and Veneto residents hospitalized in other regions, and include demographic data on the patient, admission and discharge dates, discharge status, up to six discharge diagnoses and procedures coded according to the International Classification of Diseases, 9th Revision, Clinical Modification (ICD-9-CM).

We examined hospital discharge abstracts for children aged 1–13 years and resident in Veneto Region, from 2007 to 2009. Furthermore, the data for 2008 were used for the identification of a cohort of children hospitalized for injury, and those for 2007 and 2009 were used to retrieve information on the health service utilization in the year before and after injury occurrence, respectively.

### Injury case identification

Injury has been defined as any condition identified by the ICD-9-CM codes 800–999, excluding late effects of injury (ICD-9-CM codes 905–909) and complications (ICD-9-CM code 995–999) as principal diagnosis. Whenever children had several hospital admissions coded as injury in the year of reference, 2008, the first admission was designated as the index case record.

Furthermore, the number of severe and not severe injuries have been computed, defining severity according to the Injury Severity Score (ISS) [9]. Injuries can be categorized into three different severity groups, [10] these being minor (ISS 1–8), moderate (ISS 9–15), and severe (ISS 16–75).

For the purpose of the present work, the ISSs were generated from the ICD-9-CM codes using the ICDMAP-90 software, which assigns the ISS based on ICD-9-CM discharge diagnosis codes. Hospitalizations with injury diagnoses that ascribe injuries to an unspecified body region could not be given an ISS, because ISS is anatomically based. For this reason ICDMAP-90 is unable to assign an ISS to injuries with the following ICD-9-CM diagnosis codes: 930–939 (effects of foreign body entering through orifice), 958 (certain early complications of trauma) and 960–994 (poisoning, toxic effects and unspecified effects of external causes).

### Health service utilization

The cohort of index injury cases was individually matched with all hospital discharge abstracts for 2007 and 2009 on the basis of an unique identity code.

To address possible confounding of pre-existing health conditions, the outcome was defined at the injured subject level as the difference in health service use, in terms of total hospital admissions and cumulative LOS, in the 12 months following the injury (hospitalization for index injury included), and this was compared to the year preceding the injury occurrence.

### Statistical analysis

We computed hospitalization rates - per 100,000 resident population - by gender, age class (preschool, 1-5 yrs, vs. school-aged 6- 13 yrs), and injury type. The effect of individual-level demographic and clinical information on the additional inpatient days was estimated through a linear regression model. The multivariable model included age, gender, ISS score, ICD subchapter and an interaction term between gender and ICD subchapter. Continuous independent variables were entered into the model without any transformation or cutting-off. Nonlinearity was assessed by Wald test comparing higher-order models with that including only linear terms. In case of nonlinearity, a restricted cubic spline was used to model a nonlinear effect of the covariate [11].

## Results

Hospitalization rates for injury are higher in males, with similar numbers in preschool and school-aged children, than in females, where a 26% decrease is registered in the older age class (Table 1).

**Table 1:** Hospitalization rates by injury type and gender in preschool and school-aged children; Veneto Region, 2007-2009

	**Rate per 100,000 population**			
	**Males**		**Females**	
**Injury (ICD-9CM subchapters)**	**1-5 yrs**	**6-13 yrs**	**1-5 yrs**	**6-13 yrs**
Intracranial injury (850–854)	108.2	85.1	83.2	40.0
Fracture of upper limb (810–819)	112.9	249.9	87.4	122.3
Fracture of lower limb (820–829)	61.2	84.6	30.1	46.8
Contusion with skin intact (920–924)	23.6	19.7	15.3	8.3
Fracture of skull (800–804)	25.9	38.5	16.2	14.3
Toxic effects of substances (980–989)	31.2	6.9	16.8	6.0
Complications and unspecified injuries (958–959)	24.8	31.5	25.1	13.9
Poisonings (960–979)	32.0	2.6	18.3	2.9
Burns (940–949)	34.8	6.6	26.9	4.4
Open wound of head, neck, and trunk (870–879)	56.2	16.4	34.8	10.4
Effects of foreign body entering orifice (930–939)	50.6	12.6	40.4	7.9
Internal injury of thorax, abdomen, and pelvis (860–869)	9.2	20.6	4.7	7.9
Open wound of upper limb (880–887)	20.6	12.9	10.0	4.4
Sprains and strains (840–848)	4.5	12.2	3.0	6.2
Fracture of neck and trunk (805–809)	0.3	9.8	1.2	7.2
Dislocation (830–839)	1.7	5.8	2.4	6.8
Open wound of lower limb (890–897)	4.7	6.2	3.5	3.1
Other unspecified (990–994)	5.8	2.0	0.9	1.0
Superficial injury (910–919)	5.3	4.7	5.6	2.3
Injury to nerves and spinal cord (950–957)	2.8	5.1	2.7	1.5
Injury to blood vessels (900–904)	0.0	0.5	0.3	0.0
Crushing injury (925–929)	3.1	2.2	3.0	1.5
**Total**	619.2	636.6	431.8	319.4

The main determinants of such different age-related pattern by gender are a lower rate of intracranial injury in females, and a higher rate of upper limb fractures among males. Some severe injury types increase in older children (internal injury of thorax/abdomen, fractures of neck and trunk, and - limited to males - fractures of skull), as well as lower limb fractures. By contrast, burns, effects of foreign body entering orifice, poisonings, and toxic effects of substances show dramatically lower rates with age in both genders, with males closing the gap with females for the latter two categories.

The subset of 2883 children hospitalized for injury in 2008 was included in the analysis of health services utilization. Boys represented approximately two-thirds of the cohort (63.5%) and the median age was 7 years (interquartile range: 7 years).

The injured cohort had 3233 additional hospital admissions in the year following injury, totalling 8550 additional inpatient days (Table 2). Therefore, additional 3 days/child of hospital stay are attributable to injury occurred in the last 12 months. In 2008, the median LOS for the index injury hospitalization was 2 days (interquartile range, 1 day).

**Table 2:** Number of additional inpatient admissions and days of stay in the 12 months post-injury, by International Classification of Diseases (ICD)-9 subchapters and injury severity score (ISS) of the index injury

	**Number of subjects**	Additional inpatient*	
**Injury (ICD-9CM subchapters)**		**Accesses**	**Days**
Intracranial injury (850–854)	483	510	1.586
Fracture of upper limb (810–819)	912	1028	1.776
Fracture of lower limb (820–829)	347	434	1.247
Contusion with skin intact (920–924)	82	106	324
Fracture of skull (800–804)	136	151	674
Toxic effects of substances (980–989)	79	100	166
Complications and unspecified injuries (958–959)	127	145	287
Poisonings (960–979)	69	71	179
Burns (940–949)	80	85	762
Open wound of head, neck, and trunk (870–879)	140	139	229
Effects of foreign body entering orifice (930–939)	122	126	211
Internal injury of thorax, abdomen, and pelvis (860–869)	61	61	377
Open wound of upper limb (880–887)	71	94	263
Sprains and strains (840–848)	38	40	89
Fracture of neck and trunk (805–809)	27	32	85
Dislocation (830–839)	16	16	43
Open wound of lower limb (890–897)	32	31	161
Other unspecified (990–994)	13	17	40
Superficial injury (910–919)	18	17	32
Injury to nerves and spinal cord (950–957)	14	13	−51
Injury to blood vessels (900–904)	2	3	49
Crushing injury (925–929)	14	14	21
**ISS score**			
Minor (1–8)	2172	2401	5319
Moderate (9–15)	97	146	570
Severe (≥16)	57	73	680
N.A.	557	613	1981
**Total**	2883	3233	8550

*: difference in total hospital admissions (accesses) and cumulative length of stay (days) in the 12 months after and before the index injury occurrence.

Minor injuries are responsible for most of the total injury burden (75%) and represent 62% of the additional inpatient days. Moderate and severe injuries, corresponding to 3% and 2% of all injuries, account for 14% of additional inpatient days, probably reflecting a major need of care immediately after the injury occurrence.

**Figure 1:**
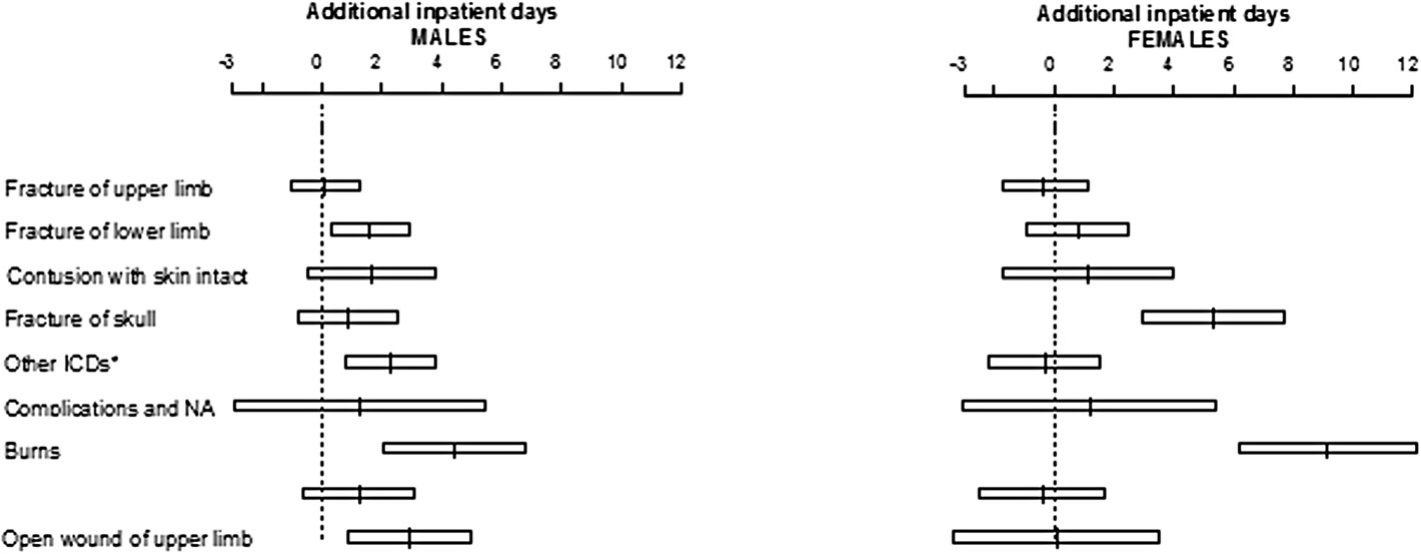
**Effects and 95% confidence intervals of ICD subchapter (reference: Intracranial injury (850–854)) on the outcome for males (on the left hand side panel) and females (on the right hand side panel).** * Other ICDs: fracture of neck and trunk (805–809), dislocation (830–839), sprains and strains (840–848), internal injury of thorax, abdomen, and pelvis (860–869), open wound of lower limb (890–897), injury to blood vessels (900–904), superficial injury (910–919), crushing injury (925–929), effects of foreign body entering orifice (930–939), injury to nerves and spinal cord (950–957), poisonings (960–979), toxic effects of substances (980–989), other unspecified (990–994).

The interaction term between gender and ICD subchapter was significant (p-value < 0.001), thus revealing that the effect of the type of injury on health resources utilization is modified by gender. Therefore, adjusted effects of ICD subchapter on the additional inpatient days are shown in Figure 1, separately for males and females.

The fracture of skull (ICD 800–804), compared to intracranial injury (ICD 850–854) leads to an increase of 5.35 (95% CI: 2.97-7.73) on the mean number of additional inpatient days among females whereas no significant effect is observed among males (0.86 days, 95% CI: -0.81-2.53). Burns (ICD 940–949) lead to a higher increase in mean additional days in females (9.14 days, 95% CI 6.16-12.13) than in males (4.40 days, 95% CI 2.02-6.79).

Age is significantly associated with the outcome. For example at 11 years of age the mean additional days increases of 0.63 (95% CI: 0.09-1.16) with respect to a 4 years old child.

**Figure 2:**
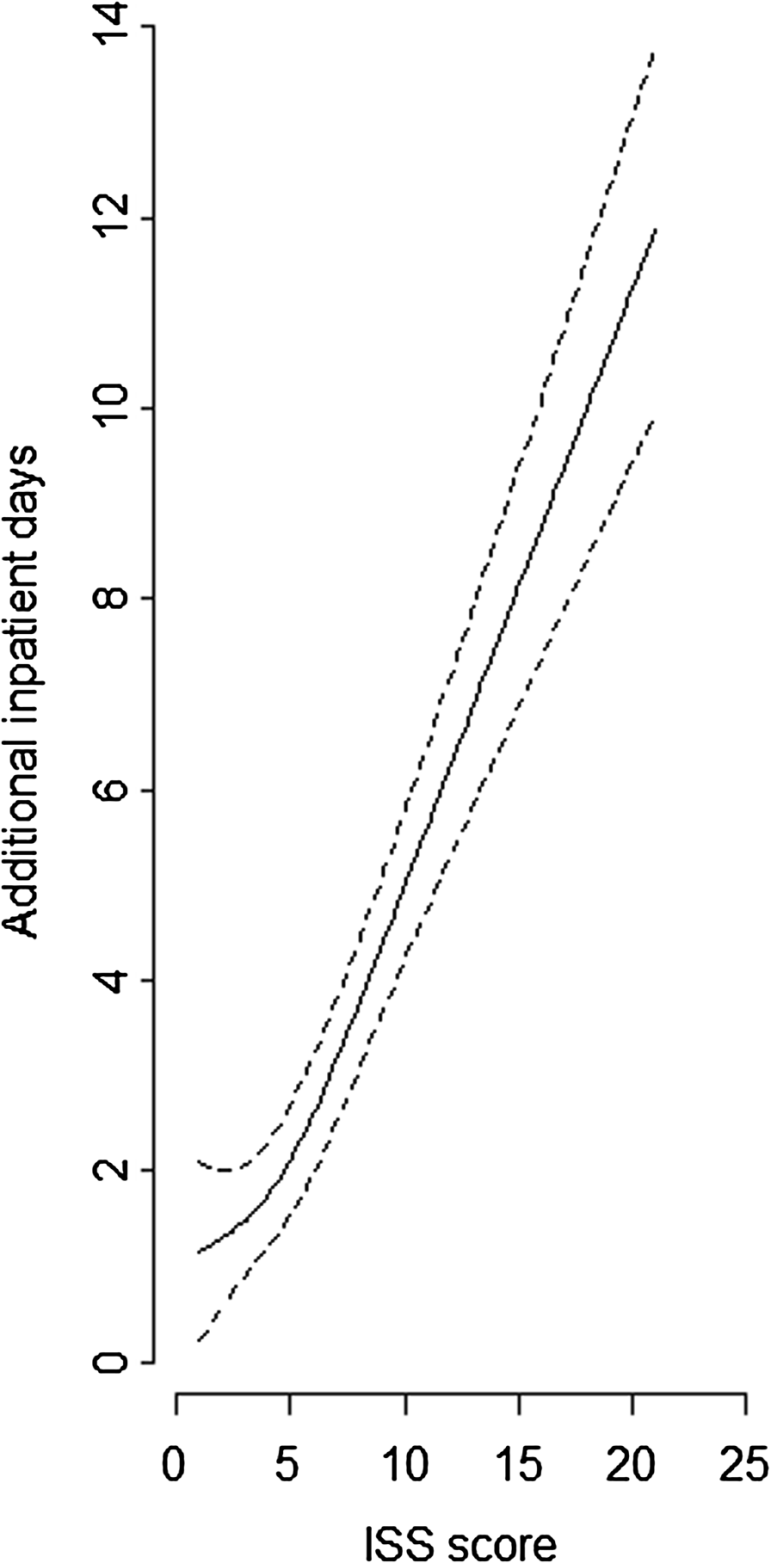
**Effect of ISS score on the outcome, estimated by restricted cubic spline.** Dotted lines indicate 95% confidence interval.

As shown in Figure 2 the effect of ISS score was nonlinear, with a stronger impact on the outcome from a score of 5 onwards. A score of 10 compared to a score of 5, leads to an increase of 2.86 (95% CI: 2.36-3.37) in mean additional days.

## Discussion

The study shows that among children <14 yrs not only injury rates, but also the impact of specific injury types on health services utilization varies with gender and age.

Overall rates indicated that boys have more injuries requiring hospitalisation than girls. The preponderance of boys in injuries has been widely reported to be an international phenomenon with boys representing more cases and higher severity levels of injuries, [2] which are associated with greater medical expenditure, longer LOS and more loss of healthy days [12]. It is possible to speculate that boys have a higher risk than girls since they are more active and adventurous in the environment, and engage in more risky behaviours than girls, with differences widening with age.

Fracture of upper limb was the most frequent injury sustained (31.6%), followed by intracranial injury (16.8%), with different age- and gender-related patterns. Children and adolescents use their upper extremities to explore their environment, assist in independent tasks of daily living, and participate in sports and play activities. For these reasons, traumatic injuries of the upper limb are extremely common and increase in the 6–13 yrs age group, in line with existing literature [13]. Additional 1.9 days of hospital stay per child are attributable to fracture of the upper limb occurred in the last 12 months.

Preschool-aged males have the highest rates of intracranial injury-related hospitalizations, comparable to those calculated by the Centers for Disease Control and Prevention (CDC) in its latest report in the United States [14]. The incidence of burns shows a similar pattern, with children 1–5 years accounting for the majority of burns and males more likely to incur injury than females. Burns requiring an hospital access are associated with the greatest number of additional inpatient days per child, about 9.5, since many hours of wound care are necessary [15].

Foreign body injuries present higher rates in preschool-aged children and imply 1.7 extra inpatient days per child. It is known that in this age range, children have a tendency to explore the environment using their mouth, but they have immature swallowing coordination and underdeveloped neuromuscular mechanisms for airway protection. Moreover older infants develop incisor teeth before the molars, which enable them to bite and detach morsels of solid food that they are unable to crush [16].

The analytical strategy for this study was based on the assumption that after controlling for pre-injury healthcare utilization, any excess in hospitalization days in the injured cohort was an outcome principally associated with the incident injury. While, to some extent, confounding by pre-existing morbidity was addressed by this study design, some unmeasured potential confounders remain. Although comprehensive administrative datasets are lacking in details regarding the circumstances surrounding injuries, they may be particularly useful for describing the overall occurrence of injury at local or regional levels, and for describing the economic implications of injury for the health care system.

While limitations to the usefulness of administrative data exist and caution must be applied in the interpretation of findings, these data are still of considerable value.

## Conclusions

These observed patterns of injury types that most impact on health resources utilization, contribute to build a clearer picture of this leading global public health problem. Along with incidence estimates and seasonal variations, [17] these results deserve more attention in planning preventive strategies and resource allocation for paediatric injuries. Modelling healthy and safe lifestyles in kindergartens and schools could be a first step to reduce injury rates.

## Competing interests

All authors declare no support from any organisation for the submitted work; no financial relationships with any organisations that might have an interest in the submitted work in the previous three years, no other relationships or activities that could appear to have influenced the submitted work.

## Authors’ contributions

IB and DG designed the study; IB, FF and UF wrote the manuscript; FA and IB performed the statistical analysis; all authors contributed to results interpretation, read and approved the final manuscript.

## References

[B1] Polinder S Haagsma JA Toet H Brugmans MJ Van Beeck EF Burden of injury in childhood and adolescence in 8 European countries BMC Public Health 2010 39 45 10.1186/1471-2458-10-45 20113463 PMC2824737

[B2] Peden M Oyegbite K Ozanne-Smith J Hyder AA Branche C Rahman AKMF Rivara FP Bartolomeos K World report on child injury prevention 2008 Geneva: World Health Organization 26269872

[B3] World Health Organization Europe European report on child injury prevention 2008 World Health Organization Europe http://www.who.int/violence_injury_prevention/child/injury/world_report/European_report.pdf

[B4] Chandran A Hyder AA Peek-Asa C The global burden of unintentional injuries and an agenda for progress Epidemiol Rev 2010 39 110 120 10.1093/epirev/mxq009 20570956 PMC2912603

[B5] WHO The Global Burden of Disease: 2004 Update 2008 Geneva: World Health Organization

[B6] Stone DH Jarvis S Pless B The continuing global challenge of injury BMJ 2001 39 1557 1558 10.1136/bmj.322.7302.1557 11431282 PMC1120608

[B7] White HL Macpherson AK Capturing paediatric injury in Ontario: differences in injury incidence using self-reported survey and health service utilisation data Inj Prev 2011 39 33 37 21646243 10.1136/injuryprev-2011-040006

[B8] Potter BK Manuel D Speechley KN Gutmanis IA Campbell MK Koval JJ Is there value in using physician billing claims along with other administrative health care data to document the burden of adolescent injury? An exploratory investigation with comparison to self-reports in Ontario, Canada BMC Health Serv Res 2005 39 15 10.1186/1472-6963-5-15 15720709 PMC554767

[B9] Baker SP O’Neill B Haddon W Jr Long WB The injury severity score: a method for describing patients with multiple injuries and evaluating emergency care J Trauma 1974 39 187 196 10.1097/00005373-197403000-00001 4814394

[B10] McClure RJ Cameron CM Purdie DM Kliewer EV Indicators of injury burden: which types are the most important? Int J Inj Contr Saf Promot 2005 39 4 213 217 10.1080/17457300500172891 16471153

[B11] Hastie T Tibshirani R Generalized additive models 1990 London, UK: Chapman & Hall 10.1177/096228029500400302 8548102

[B12] Lao Z Gifford M Dalal K Economic cost of childhood unintentional injuries Int J Prev Med 2012 39 303 312 22708026 PMC3372072

[B13] Pitone ML Attia MW Patterns of injury associated with routine childhood falls Pediatr Emerg Care 2006 39 470 474 10.1097/01.pec.0000226869.41803.50 16871104

[B14] Faul M Xu L Wald MM Coronado VG Traumatic brain injury in the United States: emergency department visits, hospitalizations, and deaths 2010 Atlanta (GA): Centers for Disease Control and Prevention, National Center for Injury Prevention and Control

[B15] Toon MH Maybauer DM Arceneaux LL Fraser JF Meyer W Runge A Maybauer MO Children with burn injuries–assessment of trauma, neglect, violence and abuse J Inj Violence Res 2011 39 98 110 21498973 10.5249/jivr.v3i2.91 PMC3134932

[B16] Gregori D Preventing foreign body injuries in children: a key role to play for the injury community Inj Prev 2008 39 411 10.1136/ip.2008.020644 19074250

[B17] Foltran F Avossa F Fedeli U Baldi I Spolaore P Gregori D Seasonal variations in injury rates in children: evidence from a 10-year study in the Veneto Region, Italy Int J Inj Contr Saf Promot 2013 39 254 258 10.1080/17457300.2012.692691 22640025

